# Acid external and internal environment exchange the *Oreochromis niloticus* tissue immune gene expression compared to the mouse macrophage polarization model

**DOI:** 10.3389/fimmu.2022.1012078

**Published:** 2022-09-26

**Authors:** Po-Kai Pan, Tsung-Meng Wu, Hsin-Yuan Tsai, I-Cheng Cho, Hsin-Wei Tseng, Tai-Du Lin, Fan-Hua Nan, Yu-Sheng Wu

**Affiliations:** ^1^ Department of Aquaculture, National Pingtung University of Science and Technology, Pingtung, Taiwan; ^2^ Biological Sciences Division, Pacific Northwest National Laboratory, Richland, WA, United States; ^3^ Department of Aquaculture, National Taiwan Ocean University, Keelung, Taiwan

**Keywords:** fresh water, environment, *Oreochromis niloticus*, mouse macrophage model, immune response

## Abstract

The water environment plays an important role in animal physiology. In this study, we sought to evaluate the effect of the acid environment on the *Oreochromis niloticus* (Nile tilapia) internal microenvironment immune response compare to the mouse macrophage model (J77A.1). The acid environment treated mouse macrophage J774A.1 model have shown that acidic treatment is able to polarize macrophages into M2-like macrophages *via* an increase in *Ym1, Tgm2, Arg1, Fizz1*, and *IL-10* expression. Metabolic analysis of mouse macrophages (J774A.1) at pH 2 vs. pH 7 and pH 4 vs. pH 7 have been shown to promote the expression of intracellular acetylcholine, choline, prochlorperazine, L-leucine, and bisphenol A,2-amino-3-methylimidazo[4,5-f] quinolone metabolites in the M2-like macrophage. Immune gene expression of the *O. niloticus* spleen and liver treated at pH 2, 4, and 7 was shown to reduce *TNF-α*, *IL-1 β*, *IL-8*, and *IL-12* expression compared to pH 7 treatment. Immune gene was induced in *O. niloticus* following culture at pH 5, 6, and 7 fresh water environment. Taken together, we found that the acid internal environment polarizes tissues into an M2 macrophage developmental microenvironment. However, if the external environment is acid, tissues are exposed to an M1 macrophage developmental microenvironment.

## Graphical Abstract

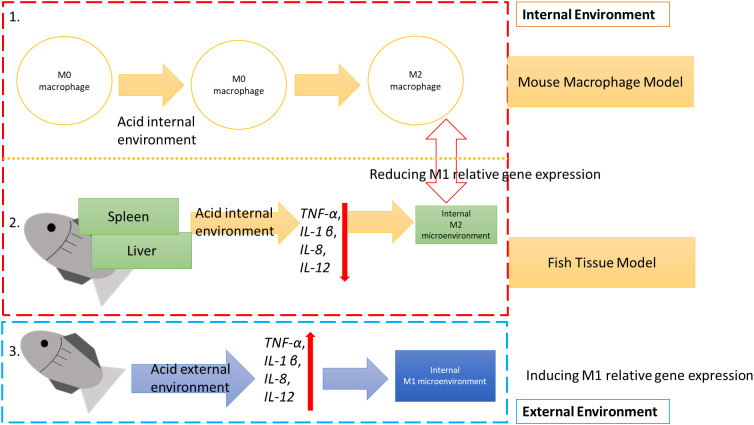

## 1 Introduction

The environment can be classically categorized as the internal and external environment. To aquatic animals, external water is important for survival, however, water acidity has become a significant global environmental problem over the past few decades. Despite this, research into the ways in which environment pH affects the internal microenvironment of aquatic animal is lacking.

Internal studies of the animal acidic environment have been shown to affect macrophages, T cells, malignant cells, adipocytes, fibroblasts, vasculatures, lymphocytes, and dendritic cells activity (1, 2). Studies have shown that an environment with a relatively low pH can suppress the anti-immune response, and therefore, maintain a suitable angiogenesis microenvironment (3). An acidic pH microenvironment can alter the function of normal immune cells (4). Lowering the environmental pH has been shown to lead to the loss of T cell function of human and murine infiltrating lymphocytes (5), to increase macrophage polarization (6), and to promote extracellular matrix-modifying enzymes (7). Further, a relatively acidic animal internal microenvironment is involved in regulating tissue metabolism through selective modification of immune cell energy metabolism.

Studies have found that tissues with a large number of macrophages, which are already known to destroy tissue infected pathogens by activating phagocytosis, ROS production, and antigen presentation are also involved in cellular factors to promote tissue recovery, growth, and angiogenesis (8, 9). Further evidence indicates that macrophages are involved in tissue healing, neovascularization, and invasion in renal cells (10). One study has also shown that macrophages participate in the immune inhibitory effects (11) by secreting cytokines, such as interleukin (IL) -10 and TGF-β, in high quantities, that attract non-cytotoxic Treg cells and Type 2 helper T cells to inhibit the differentiation and normal functions of T cells (12, 13). Tissue‐resident macrophages (M0) was known to be versatile cells polarized as a classically activated M1 or an alternatively activated M2 macrophages. M1 can be polarized by the inflammatory cytokines and microorganism‐derived molecules without the presence of lymphocytes (14). Alternative M2 already known induced by IL-4 and IL-13, characterized by increased expression of the surface markers CD206, arginase 1 (Arg1), and TGF-β involved in the tissue regeneration and anti-inflammation (15). Macrophage polarization was not only found in the mammalian system but also in the fish animals. In the redlip mullet (*Liza haematocheilus*) research, it was illustrated that *Liza haematocheilus* interferon regulatory factor 4 (*LhIRF4*) can polarize the M0 to an alternative activation as M2-like macrophage (16).

Environment factors like acidic pH, high glutathione and reactive oxygen species expression, and overexpression of some enzymes are known to participate in the environment to stimulate associated immune gene expression to polarize macrophages into M2-like macrophages (17). Previous research has also shown that an acidic microenvironment affects cell survival (18). Moreover, neutrophils suspended in bicarbonate buffer RPMI 1640 medium adjusted to acidic pH values, showed a rapid transient increase, an increase in forward light scattering properties, and upregulation of surface expression of CD18 to maintain cell survival (19).

In this study, we hope reveal the effect of the acid internal and external environment on *Oreochromis niloticus* (Nile tilapia) immune responses and compare these to the mouse macrophage J774A.1 model.

To this end, we have designed the following experiment:

### 1.1 Internal environment experiment

1. The mouse macrophage model J774.1 type was analyzed for phagocytic activity, ATP production, intracellular Ca^2+^ production, iROS production, and relative gene expression under an acid environment.
2. Analysis of intracellular metabolites of macrophages polarized under an acid environment.
3. *In vitro* experiment. The *O. niloticus* spleen and liver exposed to an acid environment medium were analyzed for immune gene expression.

### 1.2 External environment experiment

4. *In vivo* experiment. *O. niloticus* was cultured in an acid environment and subsequently analyzed for immune gene expression.

## 2 Materials and methods

### 2.1 Mouse macrophage model

#### 2.1.1 J774A.1 macrophage cell culture

A previous study showed that J774A.1 macrophages are a good model to investigate macrophage polarization (20). Hence, we selected the J774A.1 cell line to use in this study. The mouse macrophage J774A.1 cell line was obtained from the Taiwan Bioresource Collection and Research Center (BCRC number 60140). Cells were maintained in 90% Dulbecco’s modified Eagle’s medium containing 4 mM L-glutamine, adjusted to contain 1.5 g/L sodium bicarbonate and 4.5 g/L glucose + 10% (v/v) heat-inactivated fetal bovine serum with 1% (v/v) Antibiotic Antimycotic Solution (A002, HiMedia), in a humid field atmosphere of 5% CO_2_, at 37°C.

#### 2.1.2 Cellular enzyme activity

To examine the cellular dehydrogenase activity of J774A.1 macrophage cells under at pH 2, 4, and 7, J774A.1 macrophage, at a concentration of 1 × 10^6^, were seeded in a 96-well plate. Following adherence of J774A.1 macrophages, the culture media was removed and exchanged with media at pH 2, 4, and 7 for 12, 24, 48, 60, and 72 h, at 37°C, under 5% CO_2_. At the end of the incubation time, a cell counting kit-8 (CCK-8) (B34302, Bimake) and WST-1 detection kit (ab65473, Abcam) were used to examine cell survival and proliferation activity by measuring cellular dehydrogenase on a microplate reader at OD 450 nm. This experiment was replicated 3 times.

#### 2.1.3 Cellular phagocytotic activity

The phagocytic activity of J774A.1 macrophage was determined using an EZ Cell TM Phagocytosis Assay kit (Green E. coli) (K963, Biovision). Here, 1 × 10^6^ J774A.1 macrophages were seeded in a 96-well plate. Once J774A.1 macrophage had adhered, the culture media was removed and exchanged media at pH 2, 4, and 7 12 and 24 h, at 37°C, under 5% CO_2_. At the end of the incubation time, the culture media was removed, and phagocytic detection solution was added into the plate and detected at Ex/Em = 490/520. This experiment was replicated 3 times.

#### 2.1.4 ATP production activity

The ATP production of J774A.1 macrophage was determined using an ATP Colorimetric/Fluorometric Assay Kit (K354-100, Biovision). Again, 1 × 10^6^, J774A.1 macrophage were seeded in a 96-well plate. Once J774A.1 macrophages had adhered, culture media was removed and exchanged with media at pH 2, 4, and 7 media for 12 and 24 h, at 37°C, under 5% CO_2_. At the end of each incubation period the culture medium was removed, and the ATP detection solution was added into the plate and detected at Ex/Em = 535/587. This experiment was replicated 3 times.

#### 2.1.5 ROS production evaluation

ROS production were detected by a DCFH-DA (2,7-dichlorofluorescein diacetate, SIGMA) probe. Cells were incubated with 100 μM DCFH-DA in culture medium at 27°C for 30 min, and the fluorescence of the cells from each well was examined., J774A.1 macrophage cells were seeded at a concentration of 1 × 10^6^ in a 96-well plate. After adherence of J774A.1 macrophages, the culture medium was removed and exchanged with media at pH 2, 4, and 7 media, for 48 h, at 37°C, under 5% CO_2_. At the end of the treatment time, the culture medium was removed, and 100 μM DCFH-DA detection solution was added into the plate and detected at Ex/Em = 490/535. This experiment was replicated 3 times.

#### 2.1.6 Intracellular Ca^2+^ evaluation

For analysis of intracellular Ca^2+^ production, a Fluo-4 Direct Calcium Assay Kit (F10471, Thermo) was used. J774A.1 macrophage cells were seeded at a concentration of 1 × 10^6^ in a 96-well plate. Once J774A.1 macrophages had adhered to the culture plate, the culture medium was removed and exchanged with media at pH 2, 4, and 7 media, and cultured for 24 h, at 37°C, under 5% CO_2_. At the end of the treatment time, the culture medium was removed, and the detection solution was added into the plate and detected at Ex/Em = 485/525. This experiment was replicated 3 times.

#### 2.1.7 Real-time qPCR analysis

Mouse macrophage Real-time qPCR analysis of the macrophages *Ym1, Tgm2, Arg1, Fizz1* and *IL-10* was performed by Genomics Co., Ltd in Taiwan. Briefly, 1 x 10^7^ J774A.1 cells were pre-incubated in a 25T flask for 24 h at 37°C and 5% CO_2_. After pre-incubation, the medium was removed and replaced for media at pH 2, 4, and 7 and cultured for 1 and 6 h treatment. At the end of the treatment, the media was removed and collected to treat J774A.1 cells, which were scraped from 25T flask and placed in Azol^®^ Reagent. J774A.1 cells containing Azol^®^ Reagent were stored at -80°C for Real-time qPCR analysis. The following primer sequences were used:

*IL-10* (21): forward primer: CAGGGATCTTAG CTAACGGAAA, reverse primer: GCTCAGTGAATAA ATAGAATGGGAAC;
*Fizz1* (21): forward primer: CCAATCC AGCTAACTATCCCTCC, reverse primer: ACCCAGTAGC AGTCATCCCA;
*Arg1* (21): forward primer: CTCCAAGCCAA AGTCCTTAGAG, reverse primer: GGAGCTGTCATT AGGGACATCA;
*Ym1* (21): forward primer: CAGGTCTGGCA ATTCTTCTGAA, reverse primer: GTCTTGCTCATGTG TGTAAGTGA;
*Tgm2* (22): forward primer: TGTCACCAGGGATGAGAGACGG, reverse primer: TCCAAATCACACCTCTCCAGGAG
*Per1* (20): forward primer: CCAGATTGGTGGAGGTTACTGAGT, reverse primer: GCGAGAGTCTTCTTGGAGCAGTAG
*β-actin* (20): forward primer: TCACCCACACTGTGCCCATCTACGA, reverse primer: GGATGCCACAGGATTCCATACCCA

### 2.2 Metabolite analysis of the mouse macrophage model

#### 2.2.1 LC-MS metabolite analysis

To study metabolites of the J774A.1 macrophage cells at pH 2, 4, and 7, 1 × 10^6^ were seeded in a 96-well plate. Once adhered, the cell culture media was removed from J774A.1 macrophages and exchanged for media at pH 2, 4, and 7. Cells were left to culture for 24 h, at 37°C, under 5% CO_2_. At the end of this treatment time, J774A.1 macrophage were extracted with 1000 μL extraction solution (acetonitrile/methanol/water = 2:2:1). After 30 s vortex, the samples were homogenized at 35 Hz for 4 min and sonicated for 5 min in an ice-cold water bath. Next, the samples were incubated for 1 h at -20°C, and centrifuged at 13,000 rpm for 15 min at 4°C. The supernatant (421.5 μL) was transferred into a fresh Eppendorf tube and dried completely in a vacuum concentrator without heating. Subsequently, 300 μL of 50% acetonitrile was added and sonicated for 10 min in an ice-cold water bath, and centrifuged at 13,000 rpm for 15 min at 4°C. The resulting supernatant was transferred to a fresh glass vial for LC-MS analysis by BIOTOOLS Co., Ltd. (Taiwan).

Each sample (10 μL) was injected into a vanquish focused ultra-high-performance liquid chromatography (UHPLC) system coupled with an Orbitrap Elite Mass Spectrometry (Thermo, Fisher Scientific) using electrospray ionization. UHPLC parameters were set as follows: a 2.1 × 100 mm Acquity BEH 1.7 μm C18 column (Waters) was used. The column oven temperature was set at 40°C. The binary mobile phase including of deionized water containing 0.1% (v/v) formic acid as solvent A, and LC-MS grade acetonitrile with 0.1% (v/v) formic acid as solvent B. The flow rate was set at 0.25 mL/min with a linear gradient elution over 15 minutes. For the first 4 minutes, solvent B percentage was held at 0% before being linearly increased to 100% over the next 7 minutes, and kept constant for 3 minutes, before finally returning to 0% over 1 minute. To avoid any carry over effect, there was one blank injection after every sample injection, and one QC injection after every five sample injections for peak area normalization. Mass spectrometry data were collected in positive mode with a default data-dependent acquisition method, one MS full scan performed in profile mode, at 60,000 resolutions, followed by 10 data-dependent MS2 scans at 15,000 resolution. The mass scan range was set from 70 to 1000 m/z. The normalized collision energy (NCE) was 25. The spray voltage was 3.5 kV, and the capillary temperature was set at 280°C. The sheath gas was set at 30 arbitrary units and the aux gas was set at 5 arbitrary units.

### 2.3 Internal acid environment

#### 2.3.1 *Oreochromis niloticus* spleen and liver Real-time qPCR analysis

Real-time qPCR analysis of the *O. niloticus* spleen and liver *TNF-α, IL-1 β, IL-6, IL-8, IL-10*, and *IL-12* expression was performed by Genomics Co., Ltd in Taiwan.

Briefly, total of the eighteen, 30 g *O. niloticus* were euthanized and the spleen and liver was collected from each animal. Spleen and liver tissues were separated for culture in at pH 2, 4, and 7 in L-15 medium for 1 h and 6 h. At the end of the treatment, the treatment media was removed and spleen and liver samples were collected and placed in Azol^®^ Reagent. This sample mixture was then stored at -80°C for subsequent Real-time qPCR analysis. Each treatment was analyzed in triplicate. The following primer sequences were used:

*TNF-α* (23): forward primer: GAA CAC TGG CGA CAA AAC AGA;reverse primer: TTG AGT CGC TGC CTT CTA GA
*IL-12β* (23): forward primer: CAA CAG TGA CAA TCA AAT AAT TAA TAT;reverse primer: CGT TAT GTT TGT TCA CTG TGC A
*IL-8* (23): forward primer: TCG CCA CCT GTG AAG GCA T;reverse primer: TCC TTT TCA GTG TGG CAA TGA T
*IL-1β* (23): forward primer: CAG TGA AGA CCG CAA AGT GC;reverse primer: TAT CCG TCA CCT CCT CCA G
*β-actin* (23): forward primer: TCC TTC CTT GGT ATG GAA TCC;reverse primer: GTG GGG CAA TGA TCT TGA TC

### 2.4 External environment

#### 2.4.1 *Oreochromis niloticus* fish fry real-time qPCR analysis

Real-time qPCR analysis of the *O. niloticus* fish fry *TNF-α, IL-1 β, IL-8*, and *IL-12* was performed by Genomics Co., Ltd in Taiwan.

Briefly, total of the eighteen, 0.1 g *O. niloticus* fish fry were separately cultured at pH 5, 6, and 7 fresh water for 2 weeks. At the end of the culture period, the fish fry was collected and stored in Azol^®^ Reagent at -80°C for subsequent Real-time qPCR analysis. Each treatment was analyzed in six replicates. The following primer sequences were used:

***TNF-α*** (23): forward primer: GAA CAC TGG CGA CAA AAC AGA;reverse primer: TTG AGT CGC TGC CTT CTA GA
***IL-12β*** (23): forward primer: CAA CAG TGA CAA TCA AAT AAT TAA TAT;reverse primer: CGT TAT GTT TGT TCA CTG TGC A
***IL-8*** (23): forward primer: TCG CCA CCT GTG AAG GCA T;reverse primer: TCC TTT TCA GTG TGG CAA TGA T
***IL-1β*** (23): forward primer: CAG TGA AGA CCG CAA AGT GC;reverse primer: TAT CCG TCA CCT CCT CCA G
***β-actin*** (23): forward primer: TCC TTC CTT GGT ATG GAA TCC;reverse primer: GTG GGG CAA TGA TCT TGA TC

### 2.5 Statistical analysis

Scheffe and one-way ANOVA were used to analyze statistical significance. A p-value less than 0.05 was statistically significant. The results are presented as the mean ± standard deviation (SD; * p < 0.05 and ** p < 0.01). The LC-MS raw data were converted to the mzXML format using ProteoWizard and processed with an in-house program, which was developed using R and based on XCMS, for peak detection, extraction, alignment, and integration. An in-house MS2 database (BiotreeDB) was then applied in metabolite annotation. The cutoff for annotation was set at 0.5.

## 3 Results

### 3.1 Polarizing the M0 macrophage into M2-like macrophage

#### 3.1.1 Acidic microenvironment on the macrophage cell survival

**Figure 1:**
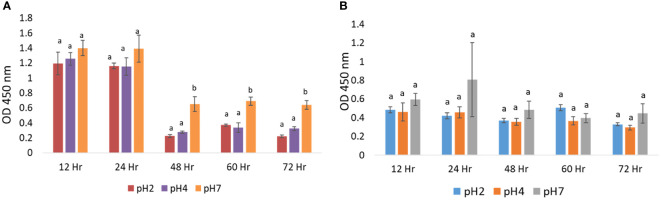
Relative cellular activation in microenvironments at pH 2, 4, and 7: **(A)** CCK-8 assay analysis of the effect of pH 2, 4, and 7 culture media on J774A.1 macrophage cell survival; **(B)** WST-1 assay analysis of the effect of pH 2, 4, and 7 culture media on J774A.1 macrophage cell survival. The a, b means with a significant difference through the Scheffe and one-way ANOVA analysis.

In cell survival experiments, J774A.1 macrophages were cultured in media at pH 2, 4, and 7 for a period of 12 to 72 h, at 37°C. There were no significant changes in the cell survival in pH 2, 4, or 7 treatment groups (p > 0.05) at 12 h and 24 h time points, using a CCK-8 detection kit, as shown in **Figure 1A**. We also used a WST-1 detection kit to confirm cell survival experimental results. J774A.1 macrophages were cultured at pH 2, 4, and 7 media for a period of 12 h to 72 h, at 37°C. With the WST-1 kit, we found that there were no significant changes in cell survival in the pH 2, 4, or 7 treatment groups (p > 0.05) at the 12 to 72 h time points, as shown in **Figure 1B**. Based on these cell survival data, we selected a treatment time of 24 h for subsequent experiments.

#### 3.1.2 Macrophage polarization phenomenon

According to the previous study, macrophage was polarized into classical activation (M1) or alternative activation (M2). To realize that the acid internal environment affected the macrophage into M1 or M2 phenomenon, we observed the macrophage activation factors as phagocytotic activity, intracellular ATP production, ROS expression and intracellular Ca^2+^.

**Figure 2:**
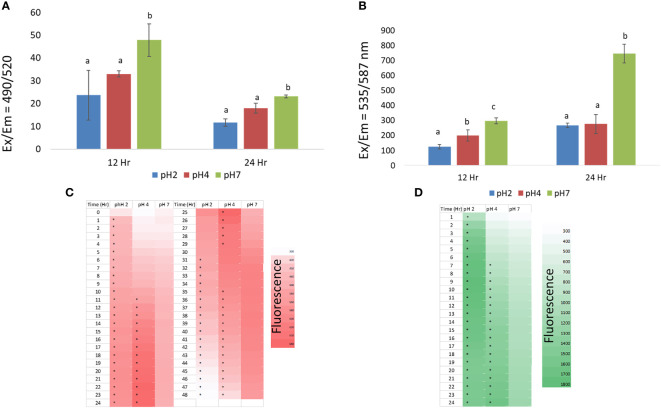
**(A)** the effect of pH 2, 4, and 7 culture media on J774A.1 macrophage phagocytotic activity; **(B)** the effect of pH 2, 4, and 7 culture media on J774A.1 macrophage ATP production; **(C)** ROS production in pH 2, 4, and 7 microenvironments, from 1 h to 48 h; **(D)** Changes in intracellular Ca^2+^ production in pH 2, 4, and 7 microenvironments, over a 1 h to 24 h time period. Representative data are expressed as the mean ± SEM; **(A–C)** means with significant change (p < 0.05). The a, b, c means with a significant difference through the Scheffe and one-way ANOVA analysis.

At the 12 h time point, we found that the pH 2 and 4 treatment groups showed some reduced phagocytotic activity compared to pH 7, but this was not significantly (p > 0.05). In addition, at the 24 h time point we found that the pH 2 and 4 treatment group showed a significant reduction in phagocytotic activity compared to the pH 7 treatment group (p < 0.05), as shown in **Figure 2A**. In this analysis, the J774A.1 macrophage cells show reduced phagocytotic activity in the pH 2 and 4 microenvironments.

At the 12 h time point we also found that the pH 2 and 4 treatment groups showed significantly reduced ATP production compared to the pH 7 treatment group (p < 0.05). At pH 4, ATP production was higher than that of the pH 2 treatment group (p < 0.05). At the 24 h time point, pH 2 and pH 4 treatment groups shows significantly reduced ATP production compared to the pH 7 treatment group (p < 0.05) as shown in **Figure 2B**. In this analysis, J774A.1 macrophage cells show reduced ATP production in the pH 2 and 4 microenvironments.

From the 1 to 13 h time points, we found that the pH 2 treatment group showed significantly enhanced ROS production compared to the pH 4 and 7 treatment groups (p < 0.05). However, the pH 2 treatment group showed a significantly reduced ROS production at the 24 to 48 h time points (p < 0.05) compared the pH 4 and 7 groups. Further, enhanced ROS production was also observed in the pH 4 microenvironment from 12 to 36 h (**Figure 2C**). These data show that ROS production is downregulated at pH 2 over the tested time scale, compared to pH 4 and 7 treatment groups. Taken together, J774A.1 macrophages show reduced ROS production in the pH 2 microenvironment. Thus, ROS production is reduced with a decrease in pH, as shown by the 32 h to 48 h data.

From 1 h to 24 h, we found that the pH 2 treatment group showed significantly increased intracellular Ca^2+^ production compared to the pH 4 and 7 groups (p < 0.05). However, the pH 2 treatment group showed a significant reduction in intracellular Ca^2+^ production, from 20 h to 24 h (p < 0.05) compared to the pH 4 and 7 groups. Moreover, enhanced intracellular Ca^2+^ production was observed in the pH 4 and 7 groups over the elapsed time (**Figure 2D**).

In conclusion, these data show that macrophage (M1) activity was reduced, likely because these cells had been polarized to the M2 phenotype.

#### 3.1.3 Effect of the acidic microenvironment on M2 macrophage relative genes

From the 1 h and 6 h time points, we found that the pH 2 and pH 4 treatment group showed a significant upregulation in the expression of M2 macrophage marker genes, including Arg1, Fizzl, Tgm2 Ym1, Per1, and IL-10.

At the 1 h time point we also observed an increase in the fold change Arg1 and Fizzl genes at pH 2 and pH 4 compared to pH 7 (control) (p < 0.05). Further, the 6 h time point also presented with an increased fold change of in Arg1 and Per 1 gene expression at pH 2 and pH 4 compared to the pH7 control (p < 0.05).

**Figure 3:**
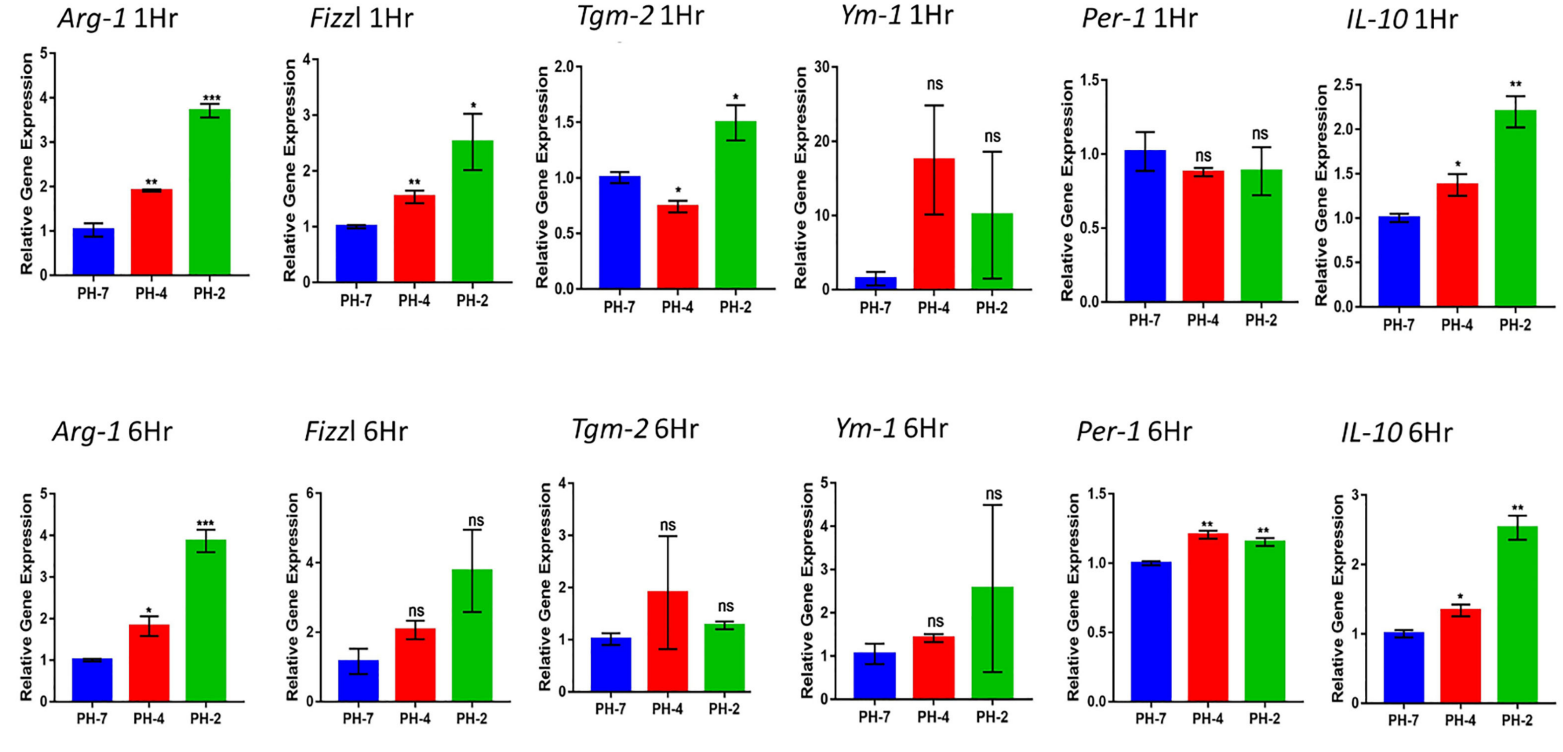
Macrophage relative expression of Arg1, Fizzl, Tgm2, Ym1, Per-1, and the M2-cytokine gene, IL-10 under the acid microenvironment. * means p< 0.05, ** means p< 0.01, *** means p < 0.005. ns means not significant.

IL-10 expression also presented with a significant increase in fold compared to the pH 7 group (p < 0.05) at both the 1 h and 6 h time points (**Figure 3**).

Taken together, we found that acidic treatment was able to polarize macrophages into M2 macrophages *via* an increase in the expression of the M2 cytokine gene, IL-10.

#### 3.1.4 Intracellular metabolite exchange of polarized macrophages in the acid microenvironment

In this study, we also analyzed the metabolite production in J774A.1 macrophages treated at pH 2, 4, and 7.

**Figure 4:**
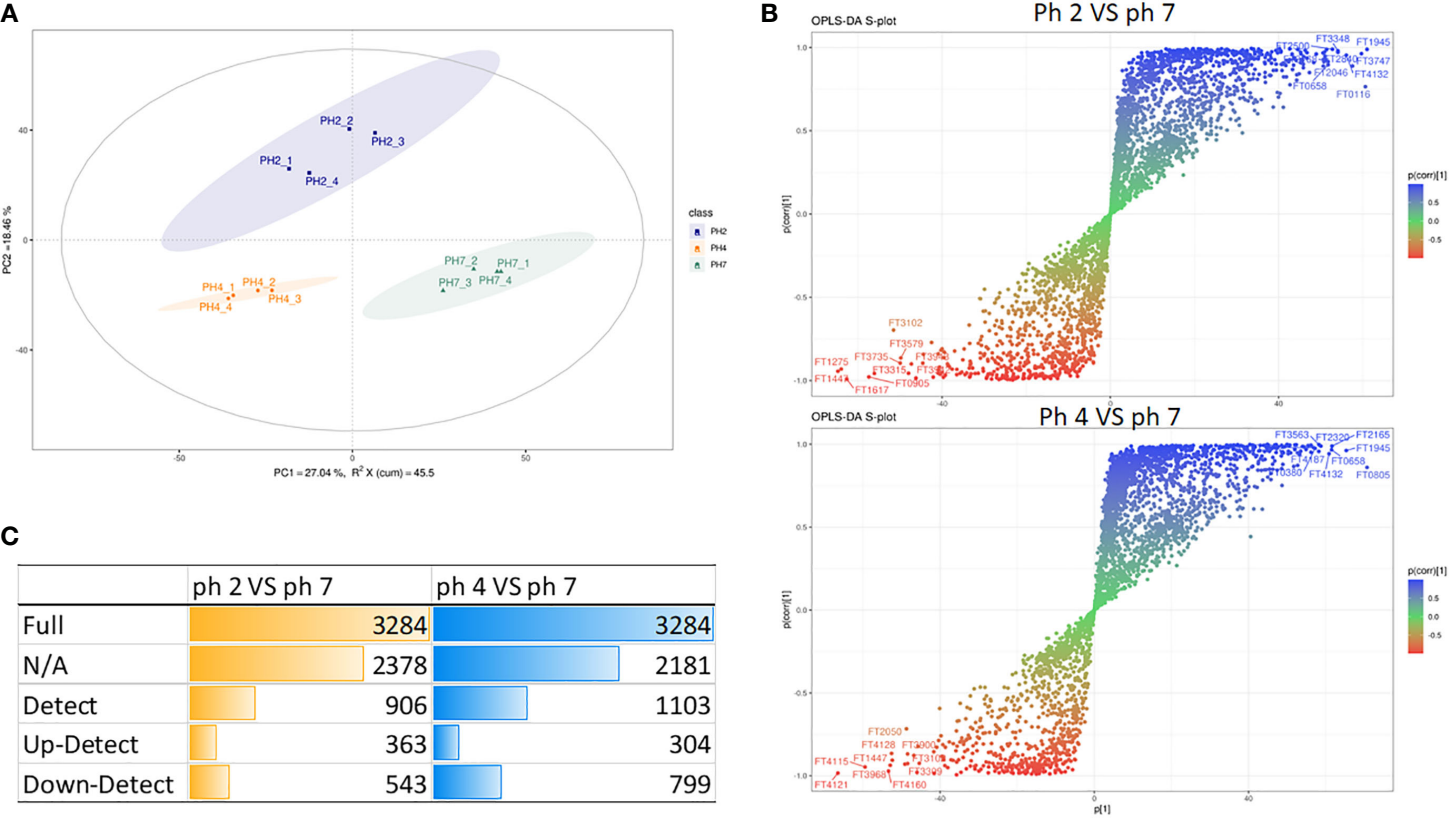
LC-MS metabolite analysis of the pH 2 vs. pH 7 and pH 4 vs. pH 7 groups: **(A)** PCA scree plots display a high resolution separation among the pH 2, 4, and 7 groups; **(B)** OPLS-DA analysis indicate that the prominent bioactive metabolites in the pH 2 vs. pH 7 and pH 4 vs. pH 7 groups are significantly changed; **(C)** the different sample analyses of the pH 2 vs. pH 7 and pH 4 vs. pH 7 groups.

**Figure 5:**
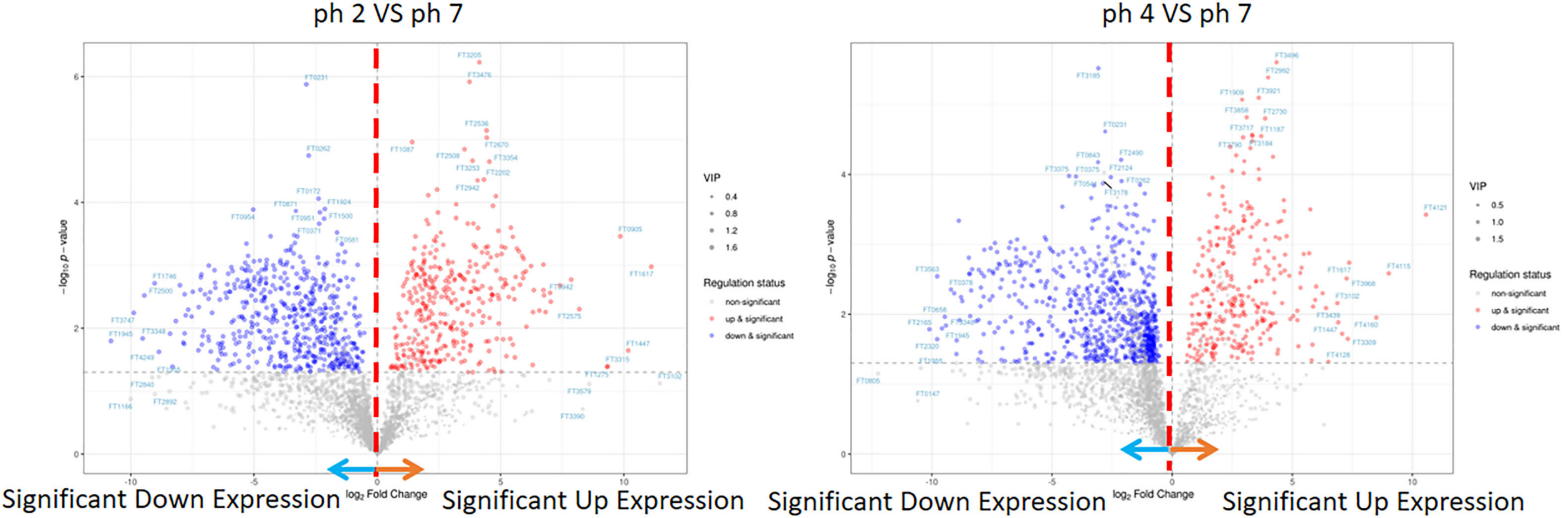
LC-MS metabolite of the pH 2 vs. pH 7 and pH 4 vs. pH 7 groups. The pH 2 vs. pH 7 and pH 4 vs. pH 7 groups were considered to be with a fold change ≥2 or a fold change.

In this analysis, the PCA scree plots displayed a high resolution separation in the pH 2, 4, and 7 groups (**Figure 4A**). OPLS-DA analysis indicated that the prominent bioactive metabolites in the pH 2 vs. pH 7 and pH 4 vs. pH 7 groups were significantly altered (**Figure 4B**). Sample analysis of the pH 2 vs. pH 7 groups showed 3284 full, 2378 N/A, and 906 detected metabolites. In the detected section, there were 363 up- and 543 downregulated metabolites. In the pH 4 vs. pH 7 groups, there were 3284 full, 2182 N/A, and 1103 detected metabolites. In the detected section, there were 304 up- and 799 downregulated metabolites as presented as **Figure 4C**. The metabolites materials of the pH 2 vs. pH 7 and pH 4 vs. pH 7 with a fold change ≥2 or a fold change ≤0.5 was presented as **Figure 5**.

The enrichment analysis (response function) of the pH 2 vs. pH 7 groups showed that three pathways were significant (i.e., “central carbon metabolism in cancer”, “chemical carcinogenesis/DNA adducts”, and “protein digestion and absorption”; **Table 1**). The pH 4 vs. pH 7 enrichment analysis (response function) showed that the “central carbon metabolism in cancer” pathway was significantly enhanced. A comparison of the pH 2 vs. pH 7 and pH 4 vs. pH 7 metabolites, revealed that “L-leucine” presented with a higher expression (pH 2 vs. pH 7, p-value 0.000345, log2 fold change 9.856, and pH 4 vs. pH 7, p-value 0.00134, log2 fold change 5.7009). The analysis data also showed a significant “bisphenol A” expression (pH 2 vs. pH 7, p-value 0.0403, log2 fold change 0.5524, and pH 4 vs. pH 7, p-value 0.00169, log2 fold change 1.6964), which was involved in the “chemical carcinogenesis/receptor activation” pathway. A comparison of pH 2 vs. pH 7 treatment groups showed an increase in “2-amino-3-methylimidazo[4,5-f] quinolone” expression (p-value 0.0163, log2 fold change 3.4931), which was involved in the “chemical carcinogenesis/DNA adducts” pathway.

**Table 1:** Enrichment analysis (response function) results for the pH 2 vs. pH 7 and pH 4 vs. pH 7 groups and types of metabolites.

ph 2 VS ph 7				ph 4 VS ph 7			
Function	Metabolics	*p*-value	log2 fold change	Function	Metaloics	*p*-value	log2 fold change
Chemical carcinogenesis-DNA adducts	2-Amino-3- methylimidazo[4,5-f]quinoline	0.016306444	3.493174987	Flavone and flavonol biosynthesis	Apigenin	0.000957962	-1.239177475
Tyrosine metabolism	3,5-Diiodo-L-tyrosine	0.041405863	-6.648580107	Chemical carcinogenesis receptor activation	Bisphenol A	0.001690417	1.696470204
Glycerophospholipid metabolism	Acetylcholine	0.002996221	6.763209171	Cephalosporins-oral agents	Cefaclor	0.004153661	-3.5183889006
Chemical carcinogenesis-receptor activation	Bisphenol A	0.04031603	0.55249117	Choline metabolism in cancer	Choline	0.001650067	3.455173781
Bile secretion	Bumetanide	0.002214466	5.105053957	Alanine, aspartate and glutamate metabolism	DL-Glutamate	0.005460247	-2.258269813
Histamine H1 receptor antagonists	Chlorcyclizine	0.00462102	-1.60068137	cAMP signaling pathway	Epinephrine	0.00705086	0.806879504
Alanine, aspartate and glutamate metabolism	DL-Glutamate	0.005935005	-3.257979173	Chemical carcinogenesis reactive oxygen species	Estradiol	0.000825131	1.96442904
Chemical carcinogenesis-DNA adducts	Ethenodeoxyadenosine	0.013271415	-7.087052017	Chemical carcinogenesis-DNA adducts	Ethenodeoxyadenosine	0.013239958	-70599195421
Manobactam biosynthesis	L-Arginine	0.001545944	-4.978182716	Monobactam biosynthesis	L-Arginine	0.001521122	-6.481211323
Tyrosine metabolism	L-Glutamic acid	0.020550335	0.767088563	Central carbon metabolism in cancer	L-Asparagine	0.007054925	-0.771070827
Central carbon metabolism in cancer	L-Glutamine	0.002139847	-2.272478425	Tyrosine metabolism	L-Glutamic Acid	0.002449081	0.946056826
Cyanoamino acid metabolism	Linamarin	0.008657569	2.172411018	Central carbon metabolism in cancer	L-Leucine	0.001341156	5.700941101
Linoleic acid metabolism	Linoleic acid	0.03449155	3.408760075	Protein digestion and absorption	L-Phenylalanine	0.003325074	-2.597108291
Central carbon metabolism in cancer	L-Leucine	0.000345851	9.856049665	N-Methyl-D-aspartic acid (NMDA) receptor antagonists	Memantine	0.006939599	-0.718441693
Protein digestion and absorption	L-Phenylalanine	0.003079225	-2.909795553	Arginine and proline metabolism	N-Acetylputrescine	0.000368568	-1.60930032
Protein digestion and absorption	L-Tyrosine	0.002791848	-3.325455947	Ion transporter inhibitors	Omeprazole	0.033916178	-1.037122165
GABA-A receptor agonists/antagonists	Primidone	0.026530428	-5.224978227	Biosynthesis of terpenoids and steroids	Paclitaxel	0.00344562	1.714121284
Antimigraines	Prochlorperazine	0.000933641	2.878733073	Tropane, piperidine and pyridine alkaloid biosynthesis	Piperine	0.004045853	1.931183672
beta-Adrenergic receptor agonists/antagonists	Propranolol	0.002888256	4.653544135	GABA-A receptor agonists/antagonists	Primidone	0.025932407	-5.870869074
				Folate biosynthesis	Sepiapterin	0.002974464	-0.792975213
				Glutathione metabolism	Spermine	0.020371738	-0.835460432
				Bile secretion	Tenofovir	0.029368278	-4.478170358
				Opioid receptor agonists/ antagonists	Tramadol	0.010523694	-0.76251471

The red mark metabolites meanto major participate in theM2-like macrophagemetabolitesproductionin the pH 2 vs. pH 7.

The bluemark metabolites mean to major participate in the M2-like macrophage metabolites production in the pH 4vs. pH 7.

The enrichment analysis (response function) revealed pH 2 vs. pH 7 and pH 4 vs. pH 7 metabolites in the J774A.1 macrophage cell line, and indicated that the acidity of the microenvironment affected these cells in the “central carbon metabolism in cancer” pathway. Moreover, a low pH was able to promote expression of intracellular metabolites, such as choline, which are involved in the “choline metabolism in cancer” pathway as presented as **Table 1**.

#### 3.1.5 Effect of an acidic environment on liver and spleen immune gene expression in *Oreochromis niloticus*

**Figure 6:**
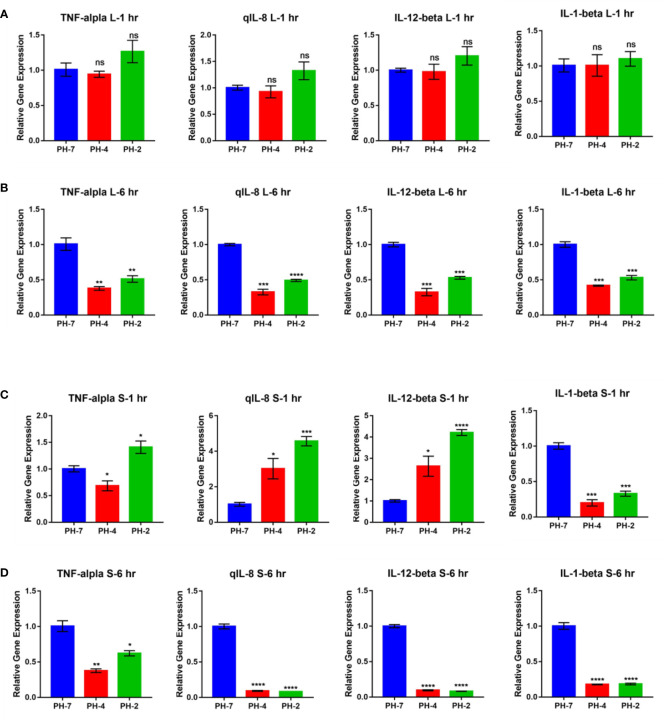
O. niloticus spleen and liver immune gene expression under pH 2, 4 and 7 at 1 hr and 6 hr. **(A)** Liver gene expression after 1 hr. **(B)** Liver gene expression after 6 hr. **(C)** Spleen gene expression after 1 hr. **(D)** Spleen gene expression after 6 hr. * means p< 0.05, ** means p< 0.01, *** means p< 0.005, **** means p< 0.001. ns means not significant.

At the 1 h time point, O. niloticus liver gene expression did not change significantly in the pH 2 and pH 4 treatment groups. Fold change analysis illustrated that TNF-α, IL-1 β, IL-8, and IL-12 gene expression was unchanged (p > 0.05) compared to the pH 7 control group at the same time point (**Figure 6A**).

In contrast, the 6 h time point presented with a reduced fold change in TNF-α, IL-1 β, IL-8, and IL-12 expression in the pH 2 and pH 4 treatment groups compared to the pH 7 control (p < 0.05; **Figure 6B**).

After 1 h, splenic gene expression showed significant changes in the pH 2 and pH 4 treatment groups. Fold change analysis showed that expression of IL-8 and IL-12 increased (p > 0.05) compared to the pH 7 treatment control, but IL-1 β expression was significantly downregulated compared to the pH 7 treatment group (p < 0.05; **Figure 6C**).

After 6 h, we observed a reduced fold change in TNF-α, IL-1 β, IL-8, and IL-12 expression in pH 2 and pH 4 treatment groups compared to the pH 7 control group (p < 0.05; **Figure 6D**).

**Figure 7:**
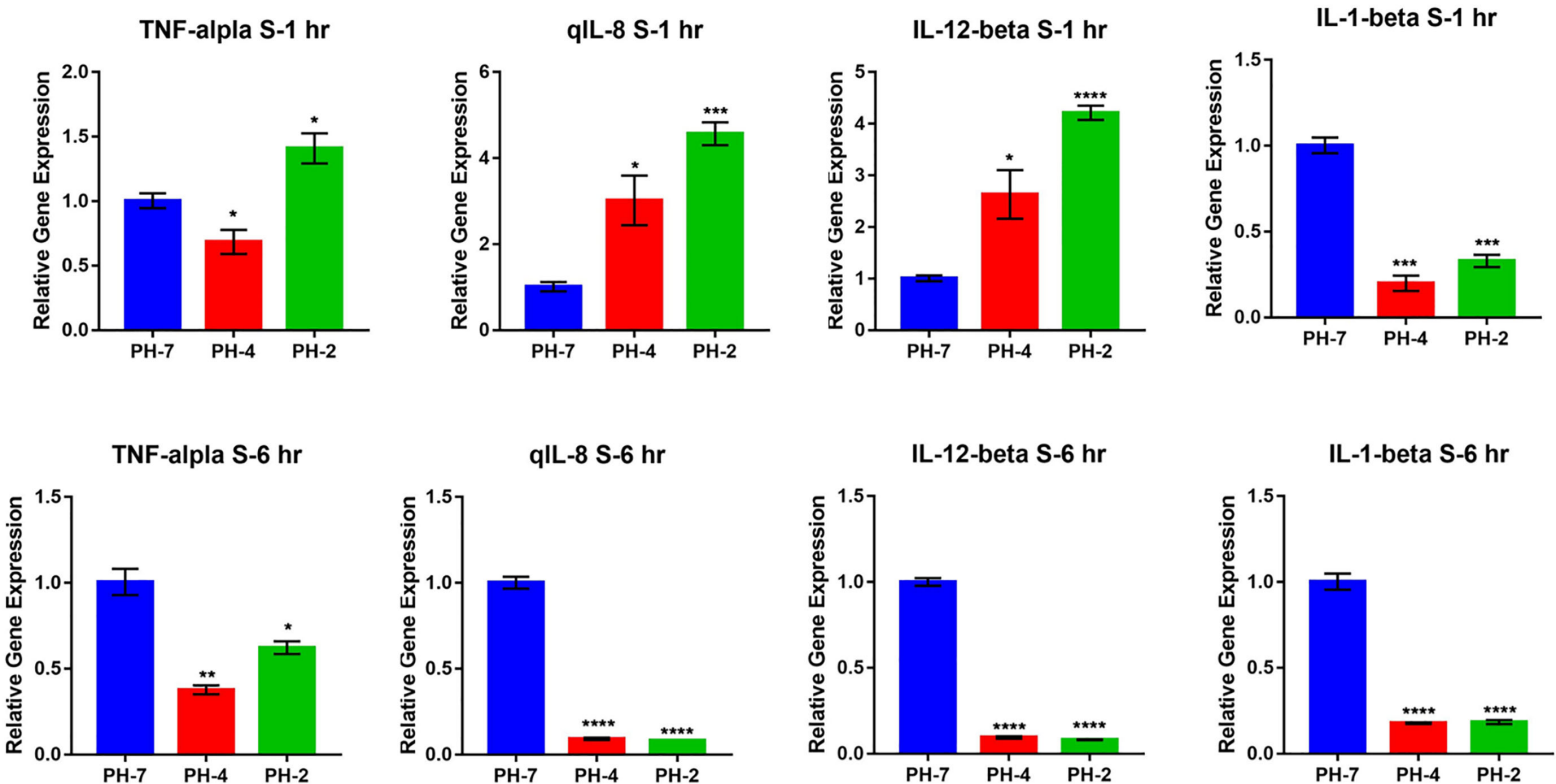
O. niloticus cultured in at pH 5, 6, and 7 for 2 weeks. Histograms showing relative immune gene expression. * means p < 0.05, ** means p < 0.01, *** means p < 0.005, **** means p < 0.001.

In the external acid experiment, we found that the acidic treatment induced O. niloticus TNF-α, IL-1 β, IL-8, and IL-12 expression for 2 weeks in culture (**Figure 7**).

## 4 Discussion

Herein, we show evidence that acidic pH-treated mouse macrophages present with upregulated expression of the M2 macrophage markers, *Ym1*, *Tgm2*, *Arg1* and *Fizz1* expression, as well as the M2 cytokine *IL-10*. Our data show that an acidic pH microenvironment promotes differentiation of M0 macrophages into M2-like macrophages. Lactate dehydrogenase-A (LDH-A) is responsible for promoting survival and proliferation of myeloid-derived suppressor cells (MDSCs). In addition, LDH-A also increases the release of pro-angiogenic factors by the murine macrophage RAW264.7 cell line through induction of vascular endothelial growth factor (VEGF) production to maintain cell survival (24).

Regarding immune responses, proinflammatory cytokines, including IL-1α, IL-1β, IL-6, IL-8, IL-18, chemokines, matrix metallopeptidase (MMP)-9, and VEGF are primarily regulated by the transcription factor nuclear factor (NF)-kB, which is active in most inflammatory conditions (25, 26). Recent studies have shown promotion of angiogenesis with the production of proangiogenic factors such as TGFβ, VEGF, PDGF, members of the FGF family, and angiogenic chemokines (27). This study, as well as our findings, show that an acidic environment downregulates M1-related functions.

Metabolite analysis showed significant L-leucine expression and reduced L-arginine expression in the pH 2 and pH 4 groups compared to the pH 7 control group. A previous study also showed that exogenous L-serine, L-valine, and L-leucine decrease the load of *K. pneumoniae* in infected lungs and increase mouse survival (28). Further, a gene level study has also demonstrated that buffering acidosis alters the activation state of macrophages, with a significant reduction in the expression of genes, including *Arg1* and *Cd206*, that are usually associated with angiogenesis (29). Research on macrophage-specific V-ATPase (ATP6V0d2), which mediates leucine-induced mTORC1 activation and polarization of macrophages, has shown that deletion of ATP6V0d2 significantly reduces splenic F4/80^+^CD11c^+^ M1 polarization but enhances F4/80^+^CD206^+^ M2 polarization upon leucine gavage (30). In addition, arginine is a strict metabolic regulator that is able to drive opposite phenotypes, depending on the metabolic pathway, and improves TNF-α, or IFN-γ and iNOS expression. NO production has been reported to be important to prevent M1 to M2 repolarization, since inhibition of iNOS induces M1 macrophages to repolarize into M2 macrophages (31). In this study, the pH 2 vs. pH 7 and pH 4 vs. pH 7 groups both presented with a downregulation of L-arginine (log2 fold change = -4.978 and -6.4812, respectively) in J774A.1 macrophages, based on LC-MS analysis. It is possible that a low pH may reduce metabolism of L-arginine, to inhibit iNOS and iROS production. Further, much like L-arginine at 24 h, iROS production in the pH 2 treatment group also decreased after 24 h.

Hormones and neurotransmitters such as catecholamines (32), acetylcholine (33), glucocorticoids, adrenocorticotropic hormone, dihydrotestosterone (34), substance P (35, 36), and adenosine (37) promote M2-like activation and inhibition of M1 activation, as previously described (38). There is evidence to show that stimulating cholinergic myenteric neurons to promote 5-HT₄R expression accelerates acetyl choline (ACh). ACh expression subsequently activates α7nAChR on activated monocytes/macrophages to inhibit inflammatory responses in the muscle layer (33). In this study, ACh was also shown to be significantly upregulated in the pH 2 vs. pH 7 group (log2 fold change, 6.7632). Taken together, we proposed that a low pH microenvironment may polarize J774A.1 macrophage into M2-like activation.

Regarding Ca^2+^ observations, a previous study indicated that sustained calcium ion release from bioceramics promoted CaSR-mediated M2 macrophage polarization (39). Moreover, there is evidence to suggest that the distinct Ca^2+^ entry channels had an important role in determining IFNγ-induced M1 or IL-4-induced M2 transition. The evidence revealed that TRPC1-mediated Ca^2+^ signaling led to M1 function, whereas Orai1-mediated Ca^2+^ signaling modulated the M2 macrophage phenotype (40). In this study, we found that intracellular Ca^2+^ production in the pH 2 treatment group increased in the early phase but subsequently declined over time. In contrast, intracellular Ca^2+^ production increased in the pH 4 and pH 7 groups. We thought that J774A.1 macrophages produced metabolites in an attempt to make the acidic culture environment more alkaline over time.

Additionally, our data show that the acidic microenvironment activates hepatic stellate cells by promoting metastasis of hepatocellular development. This was further illustrated by the fact that tumor acidification was affected to aggravate the activation of infiltrating hepatic stellate cells (HSCs) (41). Reduced O_2_ and glucose concentrations, and correspondingly increased H^+^ and lactate concentrations, occur with increasing distance from the vasculature (42). There is also evidence to suggest that tumor cells, while adapting to hypoxia which induces a change in gene expression and subsequent proteomic changes, leads to more aggressive and therapeutically resistant tumor phenotypes (43). Further, G protein-coupled receptor 132 (Gpr132) has been shown to function as a key macrophage sensor of the rising lactate in the acidic milieu to mediate the reciprocal interaction between cancer cells and macrophages (44). One previous study showed that macrophage M2 marker, including arginase-1 (*Arg1*), *Mgl-1*, Kruppel-like factor 4 (*KLF4*), and found in inflammatory zone 1 *(Fizz1*) (45). Furthermore, IL-10 has been shown to promote macrophage polarization. IL-10 is required to induce the expression of M2 markers, *Ym1* and *Fizz 1*, when macrophages are treated with DnaK (46). Furthermore, there is evidence to suggest that M2 macrophages generate anti-inflammatory cytokines, such as IL-10, and very low level of pro-inflammatory cytokine such as IL-12. Additional signatures of M2 phenotype, such as *Ym1* and *Fizz 1* have also been identified (47).

We also found that at pH 2 and pH 4, L-arginine production was reduced in macrophages (log 2 foldchange = -4.9781 and -6.4812, respectively). Taken together, we show that the acid microenvironment was able to polarize M0 macrophages to M2-like macrophages, to reduce iNOS, iROS, and phagocytotic activity. Intracellular metabolites such as ACh, choline, and prochlorperazine, which are involved in the M2-like function against the inflammatory response (in which M1 is involved). This was illustrated by the fact that an acidic pH microenvironment affects intracellular metabolite material exchange of ACh, choline, prochlorperazine, L-leucine, and bisphenol A,2-amino-3-methylimidazo[4,5-f] quinolone, among others.

A previous study showed that fish can be used as an experimental model and that fish play an important role as bio-indicators of potential risks. Furthermore, fish behavior also has a role as a biomarker in the polluted aquatic environment (48). Previous studies have shown that lysozyme activity is significantly increased in Nile tilapia cultured in acidic water at pH 4 for 2 weeks (49). In our finding, we found that in Nile tilapia, M1 relative genes (e.g. *TNF-α*, *IL-1 β*, *IL-8*, and *IL-12)* were upregulated when cultured at pH 5 and 6 for 2 weeks. Compared to the mouse macrophage experiment, we hypothesized that in an acid environment, *O. niloticus* tissues reduced their M1 immune responses to polarize the internal tissue environment into an M2 microenvironment. However, while the acid external environment the fish were polarized into M1 gene expression. Combining our internal and external acid environment data, we found that the external environment stimulates fish M1 gene expression. And, while the fish acid internal environment, M1 gene expression was downregulated in internal tissues.

The function of internal acid environment in regulation of macrophage M2 polarization has also been evidently demonstrated in mouse macrophage J774A.1. Taken together, our findings indicate the profound role of internal acid environment in modulating immune responses into M2-like micro-environment may involve in the tissue macrophage alternatively activation.

## 5 Conclusions

Data on the internal environment of the *O. niloticus* liver and spleen showed a reduction in the expression of M1 relative immune genes, including *TNF-α*, *IL-1 β*, *IL-8*, and *IL-12* in an acidic environment. The same was true for the mouse macrophage polarization model.

We also found that the acid internal environment inhibits immune responses, such as the M1 response. Further, the acid internal environment polarizes tissues into an M2 macrophage developmental microenvironment. In contrast, if the external environment was acidic, the tissue differentiated into an M1 macrophage developmental microenvironment.

## Data availability statement

The datasets presented in this study can be found in online repositories. The names of the repository/repositories and accession number(s) can be found in the article/**Supplementary Material**.

## Ethics statement

Experiment fish care and handling procedures in the present study were approved by the Laboratory Animal Center, National Pingtung University of Science and Technology.

## Author contributions

P-KP and T-MW: sample analysis and data curation. H-YT, I-CC, H-WT, and F-HN: data curation and analysis. T-DL: metabolite data and analysis writing. Y-SW: data curation, formal analysis, and article writing. All authors contributed to the article and approved the submitted version.

## Funding

This research was supported by the National Pingtung University of Science and Technology.

## Acknowledgments

The authors thank BIOTOOLS Co., Ltd. and Genomics in Taiwan for kindly supporting the analysis of the metabolites and qPCR data.

## Conflict of interest

The authors declare that the research was conducted in the absence of any commercial or financial relationships that could be construed as a potential conflict of interest.

## Publisher’s note

All claims expressed in this article are solely those of the authors and do not necessarily represent those of their affiliated organizations, or those of the publisher, the editors and the reviewers. Any product that may be evaluated in this article, or claim that may be made by its manufacturer, is not guaranteed or endorsed by the publisher.
