# Supplementary figures and images for: Acid external and internal environment exchange the Oreochromis niloticus tissue immune gene expression compared to the mouse macrophage polarization model
Source: Front Immunol. 2022 Sep 26;13:1012078. doi: 10.3389/fimmu.2022.1012078 (PMC9549756; doi:10.3389/fimmu.2022.1012078)

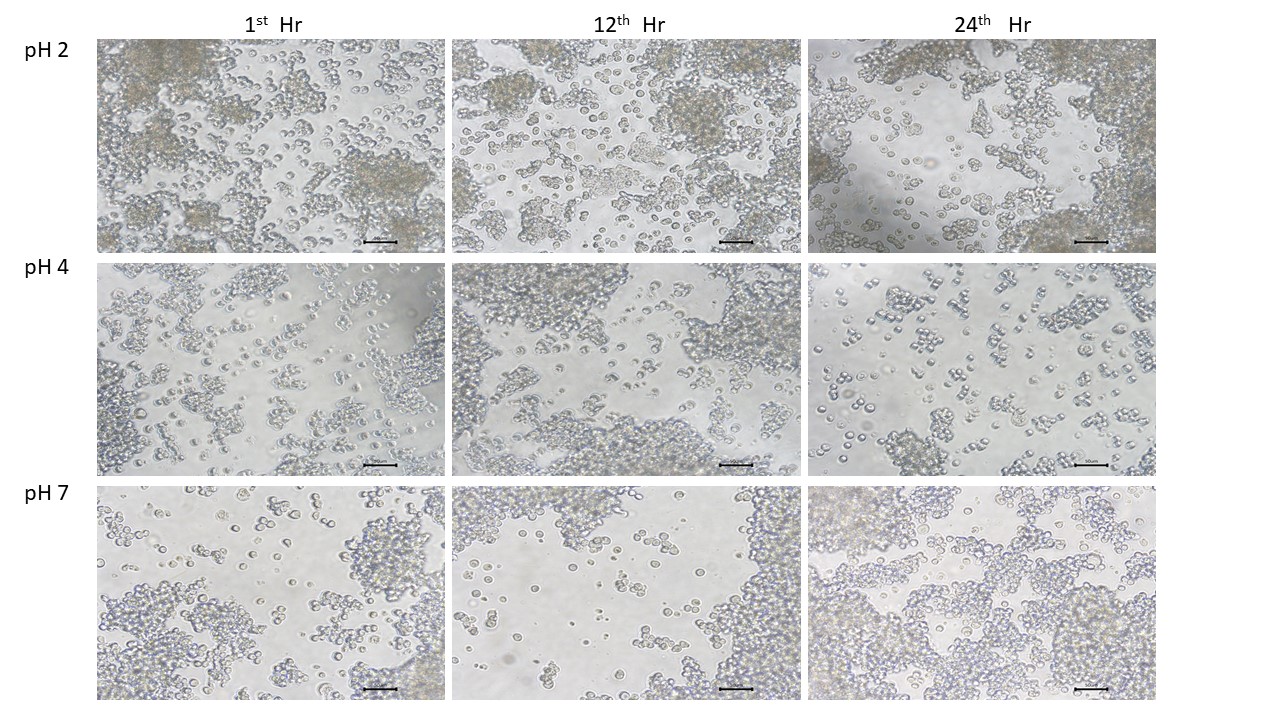

Supplement: Supplementary file 1 [file Image_1.jpg]
